# Co-inoculation with deformed wing virus and sacbrood virus affects viral and immune dynamics and synergistically increases honey bee mortality

**DOI:** 10.1371/journal.ppat.1014290

**Published:** 2026-06-25

**Authors:** Alice Mélusine Durand, Eric Dubois, Anne Bonjour-Dalmon

**Affiliations:** 1 National Research Institute for Agriculture, Food and the Environment, INRAE, UR 406 Abeilles et Environnement, Site Agroparc, Avignon, France; 2 French Agency for Food, Environmental and Occupational Health & Safety, ANSES, Sophia Antipolis, France; Duke-National University of Singapore, SINGAPORE

## Abstract

Honey bees are commonly infected with viruses, including deformed wing virus (DWV-A and DWV-B) and sacbrood virus (SBV), which cause morphological symptoms and death in developing bees and primarily asymptomatic infections in adult bees. Co-infections occur regularly in colonies, but they have rarely been studied, especially in adult bees. In this study, we co-inoculated young adult honey bees with DWV by injection (simulating vectored transmission by Varroa) and feeding them with SBV (simulating oral transmission) before reintroducing them in colonies. Through the use of optical counters and regular sampling, we tracked their survival and behaviour, and quantified the dynamics of viral loads in treated bees as well as the expression of eight immune genes involved in honey bee anti-viral immunity. Here, we show that co-inoculations of DWV and SBV synergistically increase the virulence of DWV and conditionally promote the replication of SBV. We also show that SBV may play a role in the replication of DWV in specific contexts. Finally, our results show that immune responses in adult honey bees depend on virus genotype (i.e., DWV), their relative abundance and the pre-existing natural infections before virus injection). Together, these results confirm the existence of deleterious interactions between deformed wing virus and sacbrood virus, impacting honey bee health and colony dynamics.

## Author summary

Honey bees are highly social plant pollinators that live in crowded colonies. Their population density and the high frequency of interactions between individuals favours pathogen transmission and makes colonies susceptible to pathogen outbreaks. Many viruses commonly infect honey bees, however, they are often studied as single infections. In an effort to better understand interactions between honey bees and multiple viral populations, we co-inoculated young bees with two common honey bee viruses (deformed wing virus and sacbrood virus) according to their major transmission route, released them in colonies and monitored their health and behaviour. Our findings show evidence of synergies between both viruses, as we show that a virus seemingly harmless for adult bees (sacbrood virus) may actually increase the virulence of another virus (deformed wing virus). These results highlight the importance of monitoring and studying multiple pathogens at once for a better understanding of the threat they represent to colony health and survival.

## Introduction

Bees in general, and honey bees in particular play a major role in the conservation of most terrestrial ecosystems [1]. Their plant pollinating activity is of particular importance to human life sustainability, as our agricultural production is heavily dependent on plant-pollinator interactions [2]. However, a concerning increase in annual honey bee colony mortality has been observed since the beginning of the century [3–5]. Multiples stressors and their interactions have been identified as the cause of this increasing colony mortality: habitat fragmentation, urbanization, monocultural practices, the use of pesticides, emerging parasites and pathogens [6]. As social insects living in high-density colonies, honey bee are particularly vulnerable to pathogenic infections due to facile transmission within the colony [7].

Among honey bee parasites, the mite *Varroa destructor* has become a major health issue for honey bee colonies [8]. Since the Varroa mite switched from *Apis cerana* (the Eastern honey bee) to *Apis mellifera* (the western honey bee) [9], it has gradually been introduced to most parts of the world [10–14]. Varroa reproduces by infesting capped brood and feeding on pupae haemolymph [15,16], but also feeds on adult bees’ fat tissue [16,17]. In addition to its inherent deleterious activity, Varroa can transmit various viruses to honey bees [18–20]. In particular, deformed wing virus (*Iflavirus aladeformis*, DWV) has gained much attention since the spread of Varroa. The introduction of Varroa first led to a decrease in local DWV genetic diversity [21], then a new diversification of DWV strains through competition [22,23] and recombination [24,25]. The strains most commonly found today are DWV-A and DWV-B [26]. While the DWV-A geographical expansion followed Varroa mite distribution, the context of DWV-B emergence is still not well understood [27]. Before the spread of Varroa, DWV was mainly transmitted horizontally between bees through feeding or trophallaxis [28,29], vertically from queens to eggs [30,31] and during mating [32,33]. However, transmission of DWV through the mite has greatly increased the virus pathogenicity in pupae as well as in adults [19,34,35], especially in strains (DWV-A and DWV-B) that are able to replicate within the mite [26,36,37]. DWV-B has been found to induce greater mortality than DWV-A in adult bees [19,38,39] and accumulate to higher loads [40], leading to gradual replacement of DWV-A by DWV-B [41]. Sublethal effects of DWV infections has also been reported [42–46], decreasing the foraging potential of the colony.

In the past honey bee viruses, including DWV, were primarily studied as single infections. However, honey bees are known to be infected by various viruses [47] and multiple infections commonly occur in honey bees [48–50] In recent studies, potential interactions between DWV and another honey bee virus, sacbrood virus (*Iflavirus sacbroodi*, SBV), has been suggested [45,51–53]. In Europe, SBV infections are almost ubiquitous [54–56]. Although this situation does not appear to be a critical threat to *Apis mellifera* colonies [57], the potential danger of this virus should not be overlooked. SBV dynamics and pathogenicity have not been directly affected by Varroa [20], but the mite may still indirectly impact SBV dynamics. SBV has been found to be more prevalent and higher SBV loads were found in bees from colonies infested by the mite [58], with a positive correlation between SBV loads in mites and adult bees [59]. Most SBV strains represent a major threat for *Apis cerana* colonies [60,61], but the pathogenicity of SBV can vary in *Apis mellifera* depending on SBV strains [62], suggesting that a small genomic variation can result in differential pathogenicity. Particularly high losses were experienced in *A. mellifera* in Asia with AcSBV variants [63]. Given the high mutation rate of RNA viruses [64], SBV should therefore be carefully monitored as it has the potential of becoming a serious threat to *Apis mellifera* as well.

In France, both viruses are highly prevalent and co-infections often occur [54]. Despite this, few studies have investigated co-infections with SBV and DWV [53, 65,66], and all of them have focused on honey bee eggs, larvae and pupae only. However, while symptoms related to these viruses are indeed associated with infections at early stages of development, adult bees still often carry high viral loads [67–69].

In this study, we investigated the impacts of DWV and SBV on adult honey bees as either single or co-inoculations to determine their potential of synergetic interactions. To test various scenarios of co-infections, we co-inoculated young honey bees with both viruses either simultaneously or sequentially, and used optical counters to monitor their behaviour and survival in colonies. Finally, we quantified six major honey bee viruses over time in parallel to the transcription of eight immune genes involved in honey bee anti-viral immunity [70].

## Materials and methods

### Experimental design

Our experiments were conducted between April and August 2022 and in April 2023 in France, where DWV and SBV prevalence are already highly prevalent [54]. During March 2022 and 2023, a pool of 40 adult in-hive bees from each colony in our experimental apiary was tested for the six major honey bee viruses frequently found in France (ABPV, BQCV, CBPV, DWV-A, DWV-B and SBV) by RT-qPCR. The three colonies showing the lowest viral loads from all tested colonies were each transferred into a small hive connected to an optical bee counter [71] (henceforth: ‘host colonies’). In total, four independent host-colonies were used (Table A in S1 Text). For each year of experimentation, three colonies with the lowest viral loads from the remaining colonies were selected as a source of emerging bees (henceforth: ‘donor colonies’).

The day before experimentation started, donor colony frames — from which adult bees had been removed and on which emerging brood was observed — were sampled and kept overnight in a dark incubator at 34°C. A container filled with water was placed at the bottom of the incubator to avoid air desiccation. On day 0, all the emerging bees were pooled together. For each treatment, a set of honey bees was painted on the thorax using a POSCA, while another set was marked with a 3-mm data-matrix QR code glued (Sader) on their thorax (Fig A in S1 Text). Any bee showing altered motility before or after marking was discarded.

**Fig 1:**
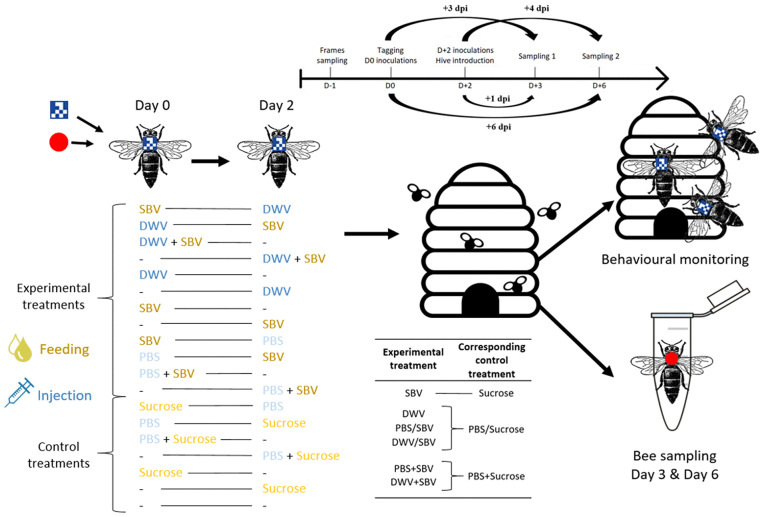
Schematic representation of the experimental protocol detailing bee treatments, their controls, and the chronological procedure. The “/” symbol represents sequential treatments on two different days while a “+” symbol represents simultaneous treatments on the same day. Sample sizes and replicates for each treatment are described in (Table A in S1 Text, A). Only one strain of DWV (either DWV-A or DWV-B) was included in each replicate (Table A in S1 Text, B).

To assess potential effects of the chronology of infections on virus interactions, inoculations were performed on day 0 after tagging and/or day 2 of each week of experimentation (Fig 1). The two viruses were either inoculated simultaneously on day 0 or 2, or sequentially on day 0 and 2. Sets of honey bees were also inoculated with a single virus (SBV, DWV-A or DWV-B) on day 0 or 2. To avoid potential methodological biases, multiple control treatments were included. Some sets of honey bees were fed with a sucrose solution (30% w/v) on day 0 or 2. Other sets of honey bees were fed with a sucrose solution (30% w/v) and injected with a PBS solution either simultaneously on day 0 or 2 or sequentially on day 0 and 2. One set of honey bees was not treated at all. In addition to bees injected with PBS and fed with sucrose, and so as to control for potential crossed effects of an orally inoculated virus and the injection method, the experiments conducted in 2023 tested sets of honey bees that were both inoculated with SBV and injected with PBS either simultaneously on day 0 or 2 or sequentially on day 0 and 2 (Fig 1). Due to the large number of treatment groups and associated bee marking and inoculations, the addition of these last treatments was not possible without removing some other treatments. As such, we decided not to replicate the sucrose-only treatments in 2023 (Table A in S1 Text). Due to the large number of bees required, each replicate was divided up and performed over the course of two weeks, which were either consecutive or one week apart. In one replicate (colony C; Table A in S1 Text), the weather did not allow for a second week of experimentation.

After inoculations, honey bees were kept in cages (inside dimensions 10 cm x 8.6 cm x 6 cm) in a dark incubator at 31°C and saturated humidity until day 2. Each cage hosted between 40 and 70 bees and contained two feeders containing a 50% sucrose solution (w/v). After day 2 inoculations, all the bees were released into a host colony. On days 3 and 6, nine painted bees from each treatment were taken from the colony and flash-frozen on dry ice, then gathered into three pools of three honey bees and kept at -80°C until analysis. Since only one host colony per interaction was tested for treatments including both SBV inoculation and PBS injection, eighteen painted bees were sampled and gathered into six pools of three honey bees for each experiment. All the treatments and experimental procedure are summarised in Fig 1.

### Virus inoculum preparation

One inoculum of each virus (SBV, DWV-A and DWV-B) was used for the experimental inoculation of honey bees. Given the recombination potential of these viruses, we only used samples originating from Europe in these experiments. Samples were selected from archived honey bee samples (supernatants of symptomatic bees in 10 mM phosphate buffer pH 7) at the ANSES laboratory of Sophia Antipolis on the basis of their previous viral quantification [72]. The genotype of the selected DWV-A and DWV-B samples had previously been analysed by next-generation sequencing [72]. The DWV-A sample (MD-GhidighiciChisinau23_2017_H156) originated from bees collected in Moldova in 2017 and was found genetically pure. The DWV-B sample (ES-Arbeiza22_2017_H62) originated from bees collected in Spain in 2017 and contained pure DWV-B as well as recombinants containing the IRES (Internal Ribosome Entry Site [73]) from DWV-A. The SBV sample originated from bees collected at Sophia-Antipolis, France, in 2017. All viruses were propagated in white-eyed pupae that were inoculated via injection. Seven days post-injection, the pupae were individually crushed in 0.01 M potassium phosphate buffer (PB; 1 mL/pupa) and centrifuged for 10 min at 8,000 x *g* at 4°C. The supernatant was then transferred to a new tube, and centrifuged again under the same conditions. Supernatant from this second centrifugation was aliquoted and analysed for the six major honey bee viruses by quantitative reverse transcription PCR (RT-qPCR) after RNA extraction [74]. The samples were selected for use as inocula only if the quantification of the virus of interest was higher than the quantification for ABPV, CBPV, BQCV and DWV or SBV (at least 10^7^ times higher for DWV samples; at least 10^10^ for SBV samples). Aliquots were filtered through a 200 µm filter, divided in 25 µL aliquots and stored at -80°C before being used as inocula for the experiments.

### Virus inoculation

DWV or PBS were inoculated through injection (Nanoject II - Drummond Scientific, Broomall, PA, USA) between the third and fourth tergites of bees. DWV inocula were diluted in PBS so as to inject 1•10^6^ genome copies in 46 nL. For each treatment group, honey bees were quickly anaesthetized with CO_2_ and kept on ice until they were all injected. Capillaries were changed between each treatment group. In contrast, SBV was inoculated orally. SBV inocula were adjusted in a 30% (w/v) sucrose solution so as to feed 1•10^8^ genome copies in 5 µL. Honey bees were starved for 2 h before being handled individually and fed using a micropipette. Sucrose feeders were placed back in the bee cages between 30 min and 2 h after inoculation. Honey bees that endured both methods on the same day (i.e., simultaneous co-inoculation) were always injected first (DWV or PBS), then fed (SBV or sucrose) within a 2-hour time frame to avoid regurgitation during anaesthesia or injection. Inocula were diluted on day 0 for each week of experimentation and kept on ice in a refrigerator (4°C) until they were discarded after day 2 inoculations.

### Behavioural monitoring

In these experiments, a total of 7,241 bees were tagged with a data-matrix QR code (Table A in S1 Text). Host colonies were connected to an optical counter [71] composed of a camera that recorded a modified hive entrance from above. The modified entrance was composed of eight tunnels that did not allow bees to walk over one another. As the bees were marked, the real-time monitoring software was able to identify individual honey bees, record the precise time and date of detection and infer the bee’s direction (going into or out of the hive; Fig A in S1 Text). Depending on the optical counter used, between 2% and 8% of recorded images did not allow identification. From this raw data, we were able to determine the proportion of bees that were detected, the pool of available and effective foragers, the age of each bee at its onset of foraging, survival after its first foraging trip, and the duration of each trip. A bee was considered an available forager if she completed at least one successful trip outside the hive and back inside. Contrastingly, a bee was considered an effective forager if she completed at least one trip outside the hive lasting more than 10 min. The last record of a given bee was considered as its time of death.

### Viral quantification and immune gene expression

Each pool of three honey bees was crushed with a 0.8 cm tungsten bead in 900µL of Trizol (Quiazol, Qiagen) using a Tissue Lyser II (Qiagen) and centrifuged at 11,000 x *g* for 30 s. A volume of 500 µL of supernatant was then transferred to a new tube. RNA was extracted according to the manufacturer’s recommendations (RNeasy Universal Mini Kit, Qiagen). A supplementary step was added between the first and second washing buffer to eliminate residual DNA (DNase PureLink, Invitrogen) according to the manufacturer’s instructions. The quantity of extracted RNA was estimated using a spectrophotometer (Nanodrop 2000, Thermo Fisher Scientific). Each sample was adjusted in H_2_O to 500 ng/µL of RNA. Reverse transcriptions were performed from 1 µg RNA according to the manufacturer’s protocol (High-capacity RNA to cDNA kit, Applied Biosystems), using random priming. Each plate was heated to 37°C for 60 min and 95°C for 5 min in a thermal cycler (Eppendorf Mastercycler, Nexus SX5). Each well was then diluted ten times so as to obtain an estimated cDNA concentration of 50 ng/µL.

Six major honey bee viruses were quantified for each sample: DWV-A, DWV-B, SBV, ABPV, CBPV and BQCV. qPCR assays were performed in duplicate in a StepOnePlus real-time PCR system (Applied Biosystems, Life Technologies). In 96-well plates, 3 µL of diluted cDNA diluted was added to 1 µL of each primer (Table B in S1 Text) with 10 µM and 5 µL of SYBR Green Master Mix (Applied Biosystems, Life Technologies), using the following thermal program: 10 min at 95°C followed by 40 cycles comprised of 15 sec at 95°C and 1 min at 60°C; a final cycle comprised of 15 sec at 95°C, 1 min at 60°C and 15 sec at 95°C was used for melt curve determination. The quantification cycles (Cqs) were retrieved and referred to a range of seven dilutions of synthetic cDNA fragments of known quantity (Eurofins, France) to deduce target quantity per bee (Equations A in S1 Text). Two H_2_O-based negative controls were systematically added to each plate: one control from the reverse-transcription step and one qPCR control.

The transcription of eight immune genes involved in various immune pathways was also quantified, namely the RNAi pathway (*argonaute-2* and *dicer*), the Imd pathway (*relish*), the humoral immune pathway (the anti-microbial peptides (AMP) *defensin-1* and *defensin-2*), the melanisation pathway (*prophenoloxidase*), the Jak-STAT pathway (*vago*) as well as *vitellogenin*, the expression of which is involved in bee longevity and immune potency. To quantify their mRNA, a similar qPCR procedure was conducted on 384-well plated in a Biorad CFX-384 thermal cycler. The list of primers used in this quantification can be found in Table B in S1 Text. One H_2_O-based qPCR control was systematically added to each plate and for each target. PCR Cqs were retrieved and compared against two reference genes (RpL32 [75]and RpS5 [76]). Additionally, a standard sample common to all the plates was added to account for any variability between runs and used as a reference to normalize variation. For each sample, all gene transcriptions were analysed on the same plate.

### Sequence validation of PCR amplicons

Relatedness between the major DWV strains detected in samples for bees inoculated with either DWV alone or in co-inoculation with SBV as well as bees inoculated with SBV and injected with PBS was assessed by Sanger sequencing of PCR products. Sequences were amplified by means of PCR using two different sets of primers (Table B in S1 Text), targeting DWV-B or potential recombinants (DWV-Rec). Direct Sanger sequencing of both strands from PCR products was performed by Genoscreen (France). Only fully sequenced PCR products were included in the phylogenetic analyses that were performed using CLUSTAL X [77] (version 1.81). The evolutionary distances were computed using the p-distance method. To handle gaps and missing data pairwise deletion was implemented. A neighbor-joining tree was built using 31 nucleotide sequences of PCR products, with bootstrap resampling (1,000 iterations). These reference sequences from databanks were added to our dataset: AY292384 (DWV-A from the US), KX373900 (recombinant genome from France), MN565037 (DWV-B from France), KX78322 (Belgium), MT74718 (Italy), AY251269 and MN538209 (Netherlands), OL80382 (Czeck Republic), H62G1-G3 (Spain), OR497383 (Pennsylvania) and OR361551-OR361553 from North Dakota, and CEND01001000 (DWV-C from UK). Visualisation of the phylogenetic trees was performed using TREEVIEW [78] (version 1.6.6).

### Statistical analysis

All statistical analyses were performed in R v4.2.2 (RStudio build 576). In all statistical analysis and graphical representations, we pooled together bees that endured similar treatment but for which D0 and D2 were interchanged. Data from each treatment group in each dataset were tested for normality (Shapiro-Wilk test, package *‘stats’*). As no dataset followed a normal distribution, non-parametric tests were used. As recommended by the Organisation for economic co-operation and development (OECD), the proportion of bees that died before reaching their onset of foraging was expressed as per cent of their respective controls [79]. Mortality proportions were compared through a pairwise nominal independence test with Benjamini-Hochberg adjustment acting as a post-hoc chi-squared test (package *‘rcompanion’*). Uncorrected mortality proportions were used to compare treatment groups to their respective controls through chi-squared tests (package *‘stats’*). Synergies were assessed using the Bliss independence test [80]. For sequentially inoculated groups in the viral dynamics analysis over time, we chose to refer to any injection as the day of inoculation (0 dpi) when analysing DWV-A and DWV-B dynamics, and to refer to any feeding as the day of inoculation when analysing SBV dynamics. The age of onset of foraging, survival of bees as foragers and viral loads were all expressed relative to their respective controls (cf. Fig 1). For that purpose, individual experimental data points were subtracted from the geometric mean of their respective controls. Dunn’s Kruskal-Wallis multiple comparison tests were performed on calculated variables with Benjamini-Hochberg adjustment (package *‘FSA’*). Each treatment group was compared to its control group through Mann-Whitney tests (package *‘stats’*) on absolute values. Comparisons between experimental treatments in the immune gene expression analysis were made using log-2 fold change values (expressed as -ΔΔCt) through Dunn’s tests with Benjamini-Hochberg adjustment (package *‘FSA’*). Cq results were adjusted based on the standard sample common to all plates. ΔCt values were calculated by subtracting the geometric mean of the adjusted Cq for two housekeeping genes (RpS5 and RpL32) to the adjusted Cq for each analysed sample Cq (target Cq – housekeeping gene Cq). ΔΔCt values were calculated by subtracting the geometric mean of ΔCt for their respective control to the ΔCt for each analysed sample (sample ΔCt – control ΔCt). Each treatment group was compared with its respective control group through Mann-Whitney tests (package *‘stats’*) on ΔCt values.

## Results

### Virus loads and dynamics

First, we assessed the potential impact of secondary viruses (BQCV, CBPV and ABPV) that were not directly involved in our experimental treatments (Fig B in S1 Text). The proportions of positive samples for each virus were similar between experimental treatments and their controls (pairwise Chi-squared tests, *p* > 0.05). Moreover, we found similar BQCV, CBPV and ABPV loads between experimental treatments and their controls (Dunn’s tests, *p* > 0.05).

**Fig 2:**
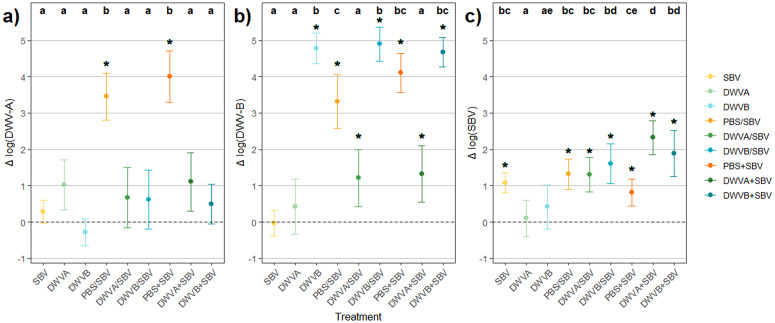
DWV-A (A), DWV-B (B) or SBV (C) loads for experimental treatments relative to their respective control groups (cf. Fig 1), irrespective of sample date. Error bars represent confidence intervals ± 95%. Letters represent statistically different groups (Dunn test). A “*” symbol marks a statistical difference between the treated group and its control group (Wilcoxon tests on absolute viral loads). A “/” symbol represents sequential manipulations over two different days while a “+” symbol represents simultaneous manipulations on the same day. All *p*-values can be found in Table C in S1 Text.

We then compared viral loads detected for DWV-A, DWV-B and SBV between each treatment, regardless of sample dates (all the *p*-values can be found in Table C in S1 Text). Untreated bees were found to carry 7.2•10^7^ DWV-A genome copies (CI95 ± 0.39 log), 4.15•10^8^ DWV-B genome copies (CI95 ± 0.42 log) and 1.11•10^9^ SBV genome copies (CI95 ± 0.48 log) (Fig D in S1 Text). Control PBS and/or sucrose inoculations did not significantly alter viral loads compared with untreated bees (*p* > 0.1). As expected, inoculation of DWV-B or SBV alone led to significantly higher loads for the respective virus compared with their respective control group, although injection of DWV-B alone led to dramatically higher DWV-B loads (Fig 2, B) than the observed SBV loads following SBV oral inoculation (Fig 2, C). However, injection of DWV-A alone did not result in significantly higher DWV-A loads compared with its control group.

Co-inoculation of both SBV and PBS led to significantly higher loads for both DWV genotypes compared with SBV inoculation alone or their control groups (Fig 2, A-B). In contrast, high DWV-A loads were not observed in groups co-inoculated with either DWV-A or DWV-B and SBV regardless of the inoculations’ time frame (Fig 2, A). Although co-inoculation with DWV-A and SBV led to significantly higher DWV-B loads compared with their control groups (Fig 2, B), DWV-B loads in bees co-inoculated with PBS and SBV were significantly higher (Fig 2, B). DWV-B loads among bees inoculated with DWV-B alone or co-inoculated with DWV-B and SBV were similar (Fig 2B).

Inoculation of DWV-A or DWV-B alone did not result in different SBV loads compared with their control groups, but simultaneous co-inoculation with either DWV genotype and SBV led to significantly higher SBV loads than simultaneous co-inoculation of PBS and SBV (Fig 2, C). Conversely, SBV loads detected in bees sequentially co-inoculated with DWV and SBV were not statistically different from those sequentially co-inoculated with PBS and SBV (Fig 2, C).

**Fig 3:**
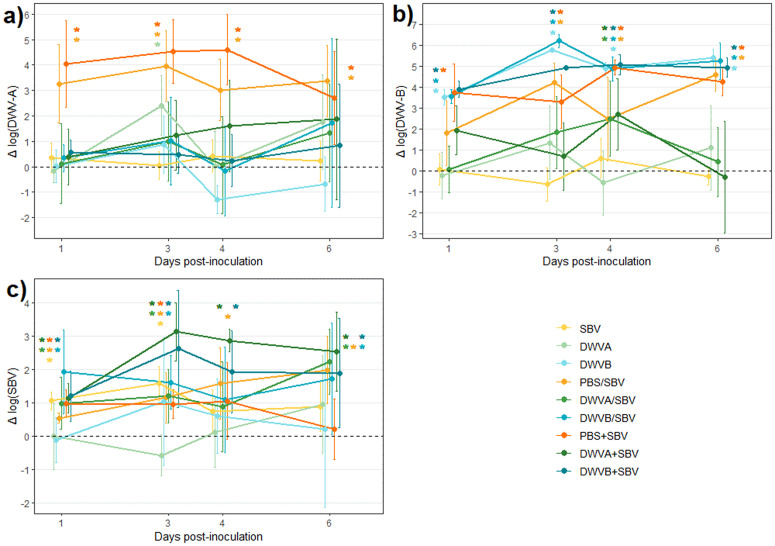
Dynamics of DWV-A (A), DWV-B (B) or SBV (C) loads over time relative to their respective control groups. Error bars represent confidence intervals ± 95%. A “*” symbol represents a statistical difference between the treated group and its control group on 6 dpi (Wilcoxon tests on absolute viral loads). A “/” symbol represents sequential manipulations over two different days while a “+” symbol represents simultaneous manipulations on the same day. Lines between points at different days post-inoculation are only present for visual clarity but do not imply interdependence between data points at different days post-inoculation, since they originate from different bees.

A temporal analysis was used to refine the impact of inoculation chronology and virus replication dynamics. All *p*-values related to this analysis can be found in the Table C in S1 Text. Here, different SBV dynamics were observed depending on whether PBS and SBV were inoculated sequentially (i.e., 24 hours apart) or simultaneously (within 2 hours). Bees inoculated with SBV alone and bees co-inoculated either simultaneously or sequentially with PBS and SBV showed significantly higher SBV loads compared with their control groups on 1 dpi and 3 dpi. However, this was only the case for bees sequentially co-inoculated with PBS and SBV on 4 dpi and 6 dpi (Fig 3, C). Compared with their control groups, we also observed significantly higher SBV loads in bees either simultaneously or sequentially co-inoculated with either DWV-A or DWV-B and SBV as early as 1 dpi. These loads remained significantly higher than their respective controls on 3 dpi, 4 dpi and 6 dpi for simultaneous co-inoculation and on 3 dpi and 6 for sequential co-inoculation (Fig 3, C).

Additionally, the previously observation of higher DWV-B loads following DWV-A and SBV co-inoculations was due to significantly higher DWV-B loads on 4 dpi only, although DWV-B loads for the two groups were again similar to their respective controls on 6 dpi (Fig 3, B). Compared with their respective controls, all SBV inoculation led to significantly higher SBV loads as soon as 1 dpi, all DWV-B inoculations led to significantly higher DWV-B loads as soon as 1 dpi. Finally, simultaneous co-inoculation with PBS and SBV led to significantly higher DWV-A and DWV-B loads as soon as 1 dpi while sequential co-inoculation with PBS and SBV led to significantly higher DWV-A loads as soon as 1 dpi but significantly higher DWV-B loads from 3 dpi and after.

Finally, the phylogenetic analysis of the viral helicase-coding sequences showed that the DWV-A and DWV-B inocula used in these experiments could be discriminated (Fig C in S1 Text). This analysis confirmed that the DWV-A strain used in this study did not replicate well in the inoculated bees. A recombinant DWV strain similar to the one found in naturally infected honey bee in Avignon (KX373900France) was found in bees inoculated with SBV and injected with PBS and in only one sample of bees co-inoculated with DWV-B and SBV. Moreover, DWV-B sequences clustering with sequences from the DWV-B inoculum were detected in the bees regardless of treatment. However, the inoculated DWV-B strains could not be discriminated from other DWV-B strains. The DWV-B sequences from our inoculum were similar to the sequences of a local DWV-B strain (MN565037France) and of DWV-B strains originating from other countries.

### Behavioural alterations and survival

**Fig 4:**
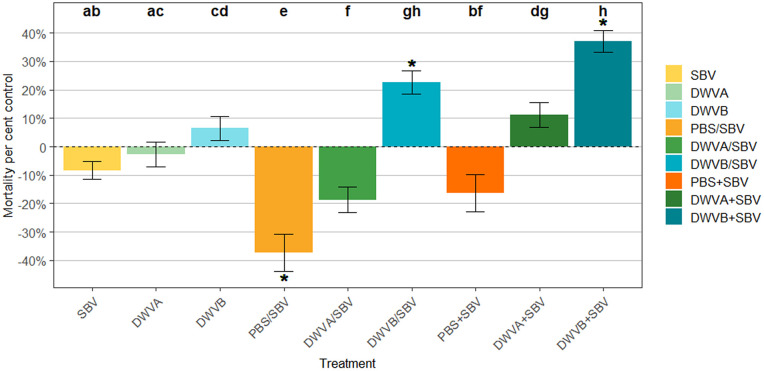
Proportions of marked bees that died before reaching their onset of foraging relative to their controls [79]. Mortality per cent control was calculated by comparing the percent of bee specifically killed by the treatment with the percent of bees that survived in the control group [59]. Letters represent statistically different groups (*p* < 0.05; pairwise nominal independence test). A “*” symbol marks a statistical difference between the treated group and its control group (*p* < 0.05; chi-squared tests on absolute proportions). Error bars represent confidence intervals ± 95% calculated from measured values. A “/” symbol represents sequential manipulations over two different days while a “+” symbol represents simultaneous manipulations on the same day. Sample sizes for each treatment can be found in Table A in S1 Text.

Among untreated bees, 47.6% (95% confidence interval (CI95) ± 4.56%) of marked bees became available foragers, suggesting that around 52.4% of them died before onset of foraging, were expelled from the hive or got lost on their first trip (Fig E in in S1 Text). Inoculation of either SBV, DWV-A or DWV-B alone did not alter the proportion of bees that became available foragers compared to their respective controls (SBV vs. Sucrose: *p* = 0.54; DWVA vs. PBS/Sucrose: *p* = 0.9; DWVB vs. PBS/Sucrose: *p* = 0.69). We observed a significantly higher proportion of bees that died before becoming available foragers in bees either simultaneously or sequentially co-inoculated with DWV-B and SBV compared with their control treatment or with either virus inoculation alone (DWVB/SBV vs. PBS/Sucrose: *p* = 0.032; DWVB+SBV vs. PBS+Sucrose: *p* = 1.17•10^-4^; DWVB/SBV vs. DWVB: *p* = 0.0095; DWVB+SBV vs. DWVB: *p* = 4.84•10^-6^; DWVB/SBV vs. SBV: p = 3.01•10^-11^; DWVB+SBV vs. SBV: p = 1.99•10^-19^ DWVB/SBV vs. PBS/SBV: *p* = 4.42•10^-24^; DWVB+SBV vs. PBS + SBV: *p* = 1.77•10^-12^; Fig 4). Moreover, observed higher mortality in bees co-inoculated with DWV-B and SBV either simultaneously or sequentially were significantly different from the additive mortality of either virus inoculated alone (*p* = 9.45•10^-20^ and *p* = 3•10^-11^, respectively; Fig E in S1 Text), indicating a synergistic effect. Sequential co-inoculation of PBS and SBV led to a significantly lower proportion of bees that died before becoming available foragers compared with its control group (*p* = 0.0194; Fig 4). Co-inoculations of DWV-A and SBV, either sequentially or simultaneously, did not alter the proportion of bees that became available foragers when compared to their respective control groups (DWVA/SBV vs. PBS/Sucrose: *p* = 0.12; DWVA+SBV vs. PBS+Sucrose: *p* = 0.3), but significantly more mortality was observed compared to bees co-inoculated with SBV and PBS (DWVA/SBV vs. PBS/SBV: *p* = 5.89•10^-8^; DWVA+SBV vs. PBS + SBV: *p* = 1.34•10^-5^; Fig 4).

**Fig 5:**
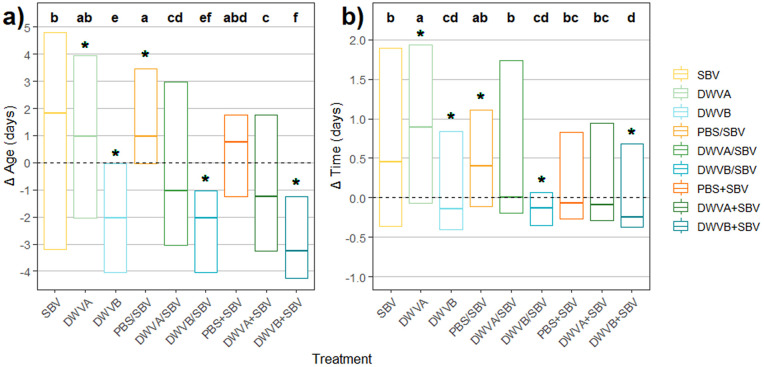
Effect of experimental treatments on the age of effective foragers. (cf. Table A in S1 Text), at their onset of foraging (A) and the time between their onset of foraging and death in effective foragers (B), both expressed as relative to their respective control groups (cf. Fig 1). For readability purposes, whiskers were removed from boxplots, which all exceeded the shown scale. Letters represent statistically different groups (Dunn’s test). A “*” symbol marks a statistical difference between the treated group and its control group (Wilcoxon tests on absolute times). A “/” symbol represents sequential manipulations over two different days while a “+” symbol represents simultaneous manipulations on the same day.

The next analysis focused solely on effective foragers (Table A in S1 Text). Untreated bees performed their first foraging trip 15.3 days (CI95 ± 1.1 days) after emergence, whereas bees injected with PBS started foraging 10.3 days (CI95 ± 0.78 days; untreated vs. PBS/Sucrose: *p* = 1.36•10^-20^; untreated vs. PBS+Sucrose: *p* = 6.84•10^-19^) after emergence (Fig E in S1 Text). Bees inoculated with DWV-B (either alone or in co-inoculation) became foragers even earlier than control bees injected with PBS (DWVB vs. PBS/Sucrose: 2.86 (± 0.94) days earlier, *p* = 9.48•10^-8^; DWVB/SBV vs. PBS/Sucrose: 3.13 (± 0.95) days earlier, *p* = 1.75•10^-8^ and DWVB+SBV vs. PBS+Sucrose: 3.58 (± 0.91) days earlier, *p* = 1.13•10^-11^; Fig 5, A) or bees co-inoculated with PBS and SBV (DWVB/SBV vs. PBS/SBV: 3.74 (± 0.87) days earlier, *p* = 4.37•10^-12^; DWVB+SBV vs. PBS + SBV: 3.46 (± 0.86) days earlier, *p* = 1.92•10^-10^; Fig 5, A). Furthermore, bees simultaneously co-inoculated with DWV-B and SBV became foragers earlier than bees inoculated with DWV-B alone (0.8 (± 0.83) days earlier, *p* = 0.022). The onset of foraging of bees inoculated with DWV-A alone and sequentially co-inoculated with PBS and SBV occured slightly later compared with their control group (DWVA vs. PBS/Sucrose: 1.1 (± 1.4) days later, *p* = 0.045; PBS/SBV vs. PBS/Sucrose: 0.6 (± 1.0) day later, *p* = 4.9•10^-4^; Fig 5, A). Regardless of the time frame of inoculations, bees co-inoculated with DWV-A and SBV became foragers significantly earlier than bees inoculated with either DWV-A alone or co-inoculated with PBS and SBV (between 1.5 and 2 days earlier, DWVA/SBV vs. DWVA: 2.14 (± 1.78) days earlier, *p* = 0.0036; DWVA/SBV vs. PBS/SBV: 1.68 (± 1.49) days earlier, *p* = 7.7•10^-5^; DWVA+SBV vs. DWVA: 2.31 (± 1.73) days earlier, *p* = 2.2•10^-4^; DWVA+SBV vs. PBS + SBV: 1.0 (± 1.37) days earlier, *p* = 0.012; Fig 5, A).

Following their onset of foraging, untreated bees foraged for 3.9 days on average (CI95 ± 0.46 days) before dying (Fig E in S1 Text). Control PBS injections did not significantly alter bee survival after they began foraging (untreated vs. PBS/Sucrose: *p* = 0.059; untreated vs. PBS+Sucrose: *p* = 0.091). Conversely, foragers inoculated with DWV-B (either alone or in co-inoculation with SBV) survived for significantly less time than their controls (DWVB vs. PBS/Sucrose: 0.8 (± 0.36) less days, *p* = 0.004; DWVB/SBV vs. PBS/Sucrose: 0.81 (± 0.372.297361) less days, *p* = 0.011; DWVB+SBV vs. PBS+Sucrose: 0.95 (± 0.43) less days, *p* = 0.0079; Fig 5, B), but co-inoculations with SBV had no effect on the survival of foragers compared with DWV-B inoculation alone (DWVB vs. DWVB/SBV: *p* = 0.9; DWVB vs. DWVB+SBV: *p* = 0.49).

### Honey bee immune gene expression

**Fig 6:**
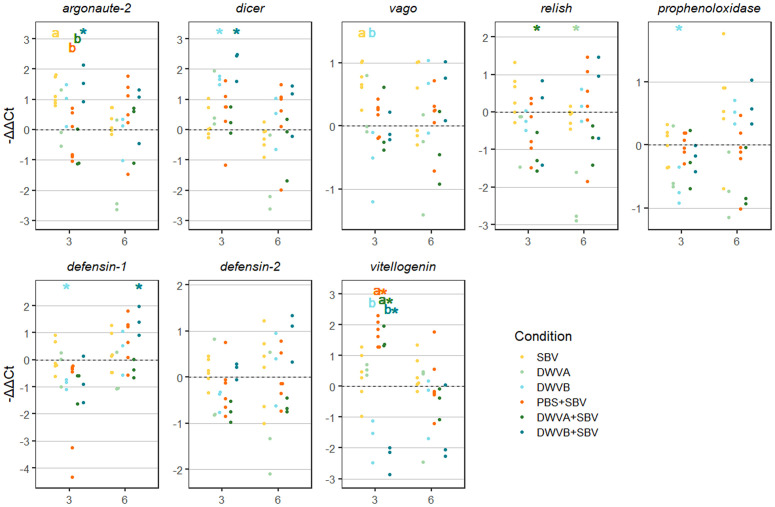
Expression of immune genes on two different days post-inoculation. Gene expression is expressed as (-ΔΔCt). Points represent single data points. Letters represent statistical differences between experimental groups (*p* < 0.05; Dunn tests on (-ΔΔCt)). A “*” symbol represents a significant difference between an experimental group and its control group (*p* < 0.05; Wilcoxon tests on ΔCt values).

In this study, we had the opportunity to analyse the immune profile of bees in different states of infection: SBV inoculation alone led to high SBV loads only; DWV-B inoculation alone led to high DWV-B loads only; DWV-A inoculation alone led to moderately high DWV-A loads at 3 dpi only (Fig 3); co-inoculation of DWV-A and SBV led to moderately high DWV-B loads and high SBV loads; co-inoculation of DWV-B and SBV led to high DWV-B and SBV loads; co-inoculation of PBS and SBV led to high DWV-A, DWV-B and SBV loads. Here, we analysed the expression of eight immune genes involved in different immune pathways (Fig 6).

Compared with their respective control group, DWV-B inoculation alone triggered a higher *dicer* expression (*p* = 0.024), and lower *prophenoloxidase* and *defensin-1* expression on 3 dpi (*p* = 0.048 and *p* = 0.024, respectively). Co-inoculation with DWV-B and SBV led to a higher expression of *argonaute-2* and *dicer* on 3 dpi (*p* = 0.024 for both comparisons), lower expression of *vitellogenin* on 3 dpi (*p* = 0.024), and higher expression of *defensin-1* on 6 dpi (*p* = 0.048). Inoculation of DWV-A alone only led to a lower expression of *relish* on 6 dpi (*p* = 0.024) while co-inoculation with DWV-A and SBV led to a lower expression of *relish* on 3 dpi (*p* = 0.024) and higher expression of *vitellogenin* on 3 dpi (*p* = 0.024). Co-inoculation with PBS and SBV only led to a higher expression of *vitellogenin* on 3 dpi (*p* = 0.0022). Bees inoculated with SBV alone expressed significantly more *argonaute-2* on 3 dpi than bees co-inoculated with DWV-A and SBV or co-inoculated with PBS and SBV (*p* = 0.038 and *p* = 0.047, respectively), and significantly more *vago* on 3 dpi than bees inoculated with DWV-B alone (*p* = 0.021). Finally, bees co-inoculated with DWV-A and SBV, and bees co-inoculated with PBS and SBV expressed significantly more *vitellogenin* on 3 dpi than bees inoculated with DWV-B, whether alone or in co-inoculation (PBS + SBV vs. DWVB: *p* = 0.012; PBS + SBV vs. DWVB+SBV: *p* = 0.007; DWVA+SBV vs. DWVB: *p* = 0.03; DWVA+SBV vs. DWVB+SBV: *p* = 0.016).

## Discussion

In this study, we investigated the effects of co-inoculation with different virus species belonging to the same viral family in adult honey bees. We showed that inoculating bees with DWV-B alone reduces the survival of foragers, and that its co-inoculation with SBV synergistically amplifies DWV-B virulence in adult bees. This synergistic effect was found in the mortality proportions of pre-foraging bees as well as in the precocious onset of foraging of bees. We also found four distinct effects of DWV and SBV co-inoculation on viral dynamics. Firstly, co-inoculation with either DWV genotype and SBV synergistically promotes SBV replication. Secondly, while PBS injections did not impact virus loads, inoculation of SBV combined with an injection-like injury promotes the replication of DWV already present in bees. But thirdly, this effect was greatly diminished when DWV-A or DWV-B was co-inoculated, suggesting competition between the DWV already present in the bee and the inoculated DWV. Finally, we found that co-inoculations often led to intensified immune responses compared to DWV inoculations alone, and that inoculation of different DWV genotypes can lead to opposite responses, even when DWV did not replicate well (i.e., DWV-A inoculations).

The viral loads and distributions of ABPV, CBPV and BQCV found in this study were similar between experimental treatments and their respective controls. This would suggest that our treatments did not have an impact on the dynamics of these three viruses. Most of our samples were positive for BQCV. Since it has been suggested that BQCV and SBV may interact [70], we cannot exclude a potential role of BQCV in the interactions we found between DWV and SBV. Since BQCV has been detected in most colonies in previous studies [59,67], its hypothetical role would most likely be maintained in natural conditions. We found an elevated proportion of samples with detectable CBPV and ABPV. However, most detected viral loads were below (or revolved around) our confidence threshold, weakening potential interpretation. We cannot fully exclude a potential role of BQCV, CBPV or ABPV in catalysing the interactions found between DWV and SBV, but the similar results found between experimental treatments and their respective controls suggest that the presence of these viruses did not skew our analysis.

In this study, we observed that co-inoculation with either DWV genotype and SBV tends to further promote SBV replication compared with virus inoculations alone. Such higher SBV replication was previously found in pupae injected with DWV [81]. This higher SBV replication was not found to be pathogenic for individual adult bees in our study (but was in another study [82]) and would not increase transmission between older adults [83]. However, it may promote higher SBV transmission between nurse bees and larvae, thus leading to higher larvae mortality and further weakening the colony. The early onset of foraging of these bees may however compensate for the potentially higher SBV transmission to larvae [82]. To address both those hypotheses, the potential of SBV to be transmitted to larvae from differentially infected adult bees, either directly from nurse bees or indirectly through the nectar collected by foragers, should be investigated in future studies.

We observed that DWV-A and SBV inoculated alone did not negatively impact honey bee survival nor induce precocious onset of foraging. Conversely, DWV-B inoculated alone did not negatively impact honey bee survival before their onset of foraging (living time spent as in-hive bees), but induced an early onset of foraging and decreased the survival of foragers. Here, we showed that either sequential or simultaneous co-inoculation of DWV-B and SBV synergistically increased the mortality of in-hive bees and simultaneous co-inoculations precipitated the onset of survivors’ foraging, potentially leading to a positive feedback loop involving an increasing proportion of early onsets of foraging in the population and general loss of in-hive population [84]. The decrease in survival of bees that became foragers was, however, similar between bees inoculated with DWV-B alone or co-inoculated with DWV-B and SBV. Thus, through higher mortality of in-hive bees and a more precocious onset of foraging, simultaneous co-exposure of bees to DWV-B and SBV may significantly reduce both the in-hive workforce and the amount of potential foragers. Furthermore, co-inoculation did not mitigate the negative impact of DWV-B inoculated alone on the survival of foragers. The negative impacts of DWV and SBV co-inoculation may have dire consequences on the colony’s capacity to forage and store resources as well as on its ability to properly carry out in-hive duties such as brood care, both being vital elements to the survival and resilience of the colony. Combined with the direct consequences of increased mortality within the colony, co-exposure of bees to DWV-B and SBV may further weaken the colony compared with exposure to one virus or the other. One study [85] compared DWV, SBV and BQCV loads and overwinter mortality in Varroa-susceptible and Varroa-resistant colonies. They found better colony survival in Varroa-resistant colonies but found similar DWV loads in Varroa-resistant and Varroa-susceptible colonies. However, SBV and BQCV loads were significantly lower in Varroa-resistant colonies than in Varroa-susceptible colonies. The results of this study are in line with our own results, and suggests that the increased virulence of DWV in the presence of SBV may indeed have tangible consequences for colony health and survival.

In our experiments, co-inoculation occurred either within a 2-hour time frame (simultaneous co-inoculation) or 48 hours apart (sequential co-inoculation). Our results suggest that the time between inoculations plays a role in the emergence of synergies between DWV-B and SBV. While both co-inoculation time-frames led to synergistically fewer available foragers for the colony (Fig 4), only the simultaneous co-inoculation further precipitated the age of onset of foraging of bees compared to DWV-B inoculation alone (Fig 5). While leading to similar levels in SBV loads on 6 days post-inoculation, we observed different dynamics in bees either simultaneously or sequentially co-inoculated (Fig 3), with more stable, high SBV loads for simultaneous co-inoculation groups. In bees subjected to SBV inoculation combined with an injection injury (PBS injection), stable, high SBV loads were only observed in the sequential inoculation scenario. In contrast, the dynamics for both DWV-A and DWV-B loads were similar between the sequential and simultaneous scenario in these bees. Therefore, we can infer that replication of DWV genotypes that were already present may not promote SBV replication, that inoculated DWV may specifically promote SBV replication when simultaneously co-inoculated, and that cuticular injury chronologically distant from SBV exposure may further promote SBV replication independently of DWV. Overall, sequential co-inoculation had a tendency to drive synergies between DWV and SBV, and simultaneous co-inoculation revealed them more clearly.

Compared with our experimental setting, bees in natural conditions are more likely to acquire lower quantities of DWV and SBV. However, they are also more likely to be exposed to both viruses more often. On the one hand, SBV is a highly prevalent virus among bees [59]. Social interactions occur frequently within the colony, as does removal of diseased larvae and pupae together with cannibalism [86,87]. This combination may lead to recurrent exposure of bees to SBV. On the other hand, our injection method aimed to mimic exposure of bees to Varroa-mediated injury and DWV transmission. Little is known about the frequency of Varroa bites, although a recent study found that Varroa mites regularly switch hosts, some of them even changing daily [88]. This would suggest that both simultaneous and sequential co-inoculations of SBV and DWV (especially the DWV-B genotype) could occur regularly in natural conditions. Furthermore, our results suggest that bites from Varroa mites free of DWV might still trigger viral replication in honey bees, as we showed that exposure to SBV combined with an injection-like injury can lead to increased replication of background DWV genotypes already present in bees. Replication of background DWV loads was even higher when DWV was not inoculated, suggesting potential competition between DWV already present and some superinfecting DWV genotypes. However, this competition may still be conditional, as superinfecting DWV-B still triggered a high increase in DWV-B when co-inoculated with SBV. As we do not know whether the observed replication of DWV-B originated from our inoculum or background DWV-B, we cannot infer whether one DWV-B genotype outcompeted the other or if an absence of competition allowed both genotypes to replicate. Although Varroa mites are known to commonly vector DWV, the viral loads of Varroa mites could be subjected to seasonality, as we detected few to no DWV-B in mites sampled in November 2022 and February 2019 in our experimental apiary, but detected comparatively higher loads in mites sampled at the end of March 2019 (Table D in S1 Text). This scenario would thus most likely occur during late autumn or early winter, although further experiments could unravel the dynamics of DWV infections in Varroa in greater detail. Finally, while the observed high DWV loads would probably not be as drastic in natural conditions than in our study, we can still expect high DWV replication in bees both injured by Varroa mites and repeatedly exposed to SBV at lower doses.

Overall, the greatest change in immune gene expression was found in bees co-inoculated with DWV-B and SBV (a significantly higher expression of *argonaute-2* and *dicer* on 3 dpi, *defensin-1* on 6 dpi and significantly lower expression of *vitellogenin* on 3 dpi, compared with its control group). Here, finding immune responses to be significantly higher than controls in co-inoculated bees while finding similar responses to controls in either virus inoculated alone is in line with another study that focused on egg transcriptomes laid by differentially infected queens [66]. We also found the greatest mortality proportions in this group of co-inoculated bees (Figs 4-5). Indeed, immune responses are known to be costly for bees [89,90]. It has also been shown that greater immune responses may be linked to higher susceptibility to pathogen replication in young bees [91]. Other authors found lower *vitellogenin* expression in DWV-inoculated bees [44,92]. In our study, *vitellogenin* expression was only found to be significantly lower in bees co-inoculated with DWV-B and SBV. Immune responses require Vitellogenin [93,94], so an elevated immune response combined with decreased *vitellogenin* expression may lead to a drastic depletion of Vitellogenin stores. As Vitellogenin levels control task allocation [95] and are linked to longevity [93], the depletion of Vitellogenin stores may explain both the higher mortality observed and the early onset of foraging of these co-inoculated bees. However, we did not find the opposite to be true, as simultaneous DWV-A and SBV co-inoculation and simultaneous PBS and SBV co-inoculation led to significantly elevated *vitellogenin* expression, but no change in survival or age of the onset of foraging was observed compared with their control group. Interestingly, unlike DWV-B replication triggered by a superinfecting DWV-B, high replication of DWV-B already present in bees did not lead to either pronounced immune responses or elevated mortality.

Additionally, multiple trends may be discussed. Firstly, the immune gene expression profiles of co-inoculated bees tended to be more similar to those of bees inoculated with DWV alone than to those of bees inoculated with SBV alone (e.g., *dicer* on 3 dpi, *vago* and *prophenoloxidase* on 6 dpi). Co-inoculation of SBV with one DWV genotype tended to lead to a qualitatively similar (higher or lower expression) but quantitatively exacerbated response compared with DWV inoculation alone (e.g., *argonaute-2*, *dicer* and *vitellogenin* on 3 dpi, *defensin-1* and *defensin-2* on 6 dpi). Secondly, when co-inoculated with SBV, DWV-A and DWV-B tended to trigger opposite responses (e.g., *argonaute-2*, *dicer*, *relish* and *vitellogenin* on 3 dpi, *vago*, *prophenoloxidase*, *defensin-1* and *defensin-2* on 6 dpi). Inoculations of DWV-A, either alone or in co-inoculation, did not trigger elevated immune responses despite this being the case for DWV-B inoculations. This could either originate from the DWV genotype or the degree of viral replication, as DWV-B replicated more than DWV-A when inoculated (Figs 2-3). However, given that immune genes expressions were only analysed in a few bees per treatment and did not extend to all treatments, our current analysis only represent a first step towards understanding immune dynamics in cases of co-inoculations. Our results call for deeper investigations especially for cases of simultaneous co-inoculations as well as cases of stimulation of background viral replication.

When observed, the RNAi pathway was activated on 3 dpi but not on 6 dpi, confirming that this pathway is an early defence system against viruses [96]. However, other studies have found RNAi gene transcription activated as late as 5 dpi [97,98]. Similarly to our results, one study [98] found that inhibition of *defensin-1* can occur in parallel to the activation of the RNAi pathway in DWV-B inoculated bees. Our study shows that there may be concurrent inhibition of the melanisation pathway, which was not the case in other studies [53,99]. Conversely, when observed, AMP production was activated on 6 dpi but not on 3 dpi, confirming that AMPs are used as a late defence system that may remain active for longer times [92]. However, our study highlights the importance of the context of infection and virus interaction in the bee immune response.

Honey bee health has become a major concern in the past decades. As research has progressed, the network of interactions between honey bee stressors has been shown to be more complex than previously thought. Here, we introduce viral interactions as a significant additional level of complexity. According to our results, SBV seem to play a role in the increased DWV virulence and replication observed since the introduction of *Varroa destructor,* despite different major routes of infection between the two viruses. Moreover, interactions between the two viruses may also favour SBV replication, potentially leading to increased frequency of co-exposure, possibly resulting in a positive feedback loop of SBV replication and transmission. Our study highlights the importance of viral interactions and the need for future research to consider the complexity of the honey bee virobiome in the study of honey bee health. However, while studying the effects of virus inoculations directly in hives is more representative of natural conditions of infection, introducing viruses in hives always comes at a risk of higher recombination events. For this reason, strains selection and the scale of the experiments must be carefully thought.

## Supporting information

S1 Text Table A in S1 Text Summary of replicates and sample sizes for each treatment in our analyses. For each treatment: number of host colonies tested, number of replicates, sample sizes for each figure and the chronology of experiments. **Table B in S1 Text Virus and immune gene primers.** Primers used for virus sequence amplification and quantification and immune gene expression analysis and their references. **Equations A in S1 Text Estimation of viral copies in each bees using three equations.** Equations used for estimating viral copies found in individual bees based in Cq values. **Fig A in S1 Text Visualisation of marked frame-bees and optical counter recording. (**a) Picture of a hive frame section including painted bees, QR-code-marked bees and the painted queen. (b) Picture of a bees passing through an optical counter tunnel. **Fig B in S1 Text BQCV, CBPV and ABPV virus detection analysis.** Graphical representations of BQCV, ABPV and CBPV viral prevalence and viral loads found in positive bees. **Table C in S1 Text Summary of *p*-values found in the viral loads quantification analysis.** A) Table showing *p*-values found in the overall viral loads analysis. B) Table showing *p*-values found in the temporal analysis of viral loads. **Fig C in S1 Text Phylogenetic tree of DWV sequences detected by PCR in experimental samples.** Phylogenetic tree of DWV sequences constructed from 1314 nucleotides-long sequences from the helicase-coding sequence. **Fig D in S1 Text DWV-A (A), DWV-B (B) or SBV (C) loads for all treatments.** Raw data showing viral loads for all treatments instead as relative to controls. **Fig E in S1 Text Data analysis originating from optical counters for all treatments.** A) Raw data showing proportions of marked bees that became effective foragers for all treatments. B) Expected and observed mortality proportions for bees co-inoculated with DWV-B and SBV. C) Raw data for the age at which effective foragers performed their first foraging flight for all treatments. D) Raw data for the time between the onset of foraging and death of effective foragers for all treatments. **Table D in S1 Text Viral loads of Varroa destructor mites collected throughout the year 2019 and 2021.** DWV-A, DWV-B, ABPV, BQCV and SBV viral quantifications performed on Varroa destructor mites collected on autumn 2021, winter 2019 and spring 2019 on the apiary of ANSES Sophia Antipolis.
(DOCX)
