# Supplementary material for: Co-inoculation with deformed wing virus and sacbrood virus affects viral and immune dynamics and synergistically increases honey bee mortality
Source: PLoS Pathog. 2026 Jun 25;22(6):e1014290. doi: 10.1371/journal.ppat.1014290 (PMC13298744; doi:10.1371/journal.ppat.1014290)
Supplement: S1 Text — For each treatment: number of host colonies tested, number of replicates, sample sizes for each figure and the chronology of experiments. Table B in S1 Text Virus and immune gene primers. Primers used for virus sequence amplification and quantification and immune gene expression analysis and their references. Equations A in S1 Text Estimation of viral copies in each bees using three equations. Equations used for estimating viral copies found in individual bees based in Cq values. Fig A in S1 Text Visualisation of marked frame-bees and optical counter recording. (a) Picture of a hive frame section including painted bees, QR-code-marked bees and the painted queen. (b) Picture of a bees passing through an optical counter tunnel. Fig B in S1 Text BQCV, CBPV and ABPV virus detection analysis. Graphical representations of BQCV, ABPV and CBPV viral prevalence and viral loads found in positive bees. Table C in S1 Text Summary of p-values found in the viral loads quantification analysis. A) Table showing p-values found in the overall viral loads analysis. B) Table showing p-values found in the temporal analysis of viral loads. Fig C in S1 Text Phylogenetic tree of DWV sequences detected by PCR in experimental samples. Phylogenetic tree of DWV sequences constructed from 1314 nucleotides-long sequences from the helicase-coding sequence. Fig D in S1 Text DWV-A (A), DWV-B (B) or SBV (C) loads for all treatments. Raw data showing viral loads for all treatments instead as relative to controls. Fig E in S1 Text Data analysis originating from optical counters for all treatments. A) Raw data showing proportions of marked bees that became effective foragers for all treatments. B) Expected and observed mortality proportions for bees co-inoculated with DWV-B and SBV. C) Raw data for the age at which effective foragers performed their first foraging flight for all treatments. D) Raw data for the time between the onset of foraging and death of effective foragers for all treatments. Table [file ppat.1014290.s001.docx]

Co-infection with deformed wing virus and sacbrood virus affects viral and immune dynamics and synergistically increases honey bee mortality

Supporting Information


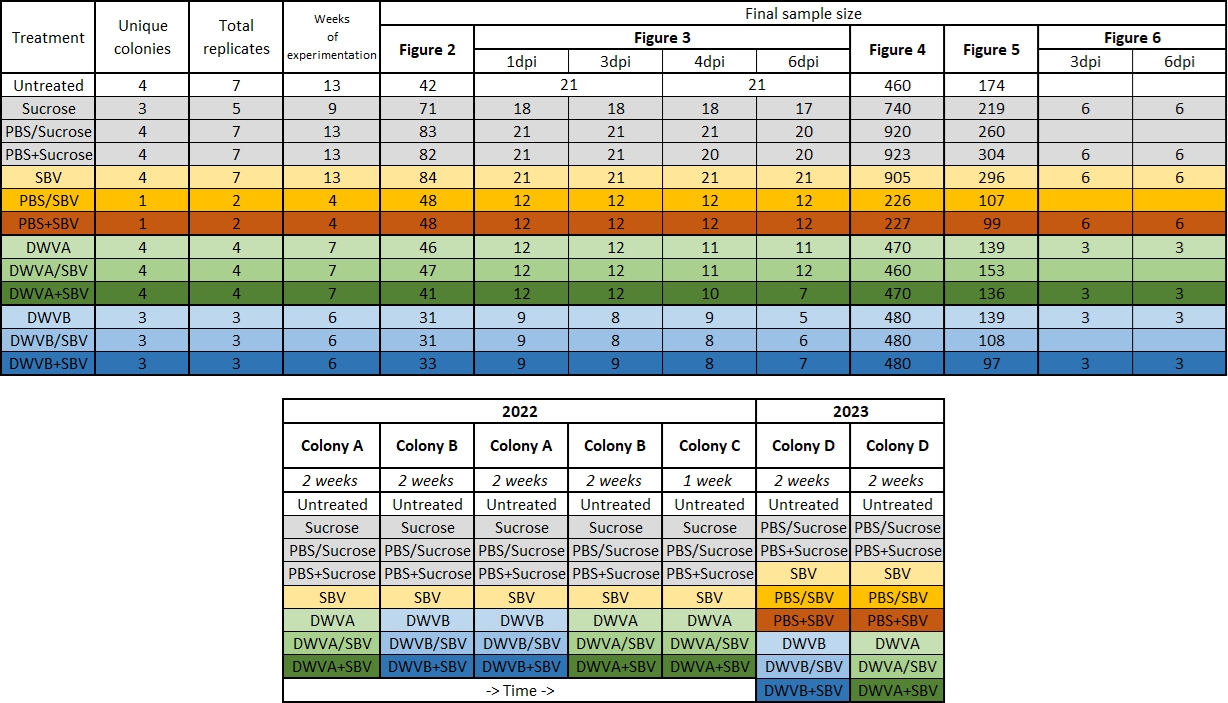


**Table A. Summary of replicates and sample sizes for each treatment in our analyses.** The first table describes the total number of colonies, replicates and bees included for each treatment. The second table describes the chronology of experiments, and which treatment was included in each replicate. For Figure 4, 5 and 6, each sample consists of a pool of three honey bees. Numbers ranging over two timepoints lacked a treatment of reference (Untreated).

| Virus / Gene | Forward primer (5’ -> 3’) | Reverse primer (5’ -> 3’) | Reference |
| --- | --- | --- | --- |
| ABPV | TCATACCTGCCGATCAAG | CTGAATAATACTGTGCGTATC | (Locke et al. 2012) |
| BQCV | AGTGGCGGAGATGTATGC | GGAGGTGAAGTGGCTATATC | (Locke et al. 2012) |
| CBPV | CAACCTGCCTCAACACAG | AATCTGGCAAGGTTGACTGG | (Locke et al. 2012) |
| DWV-A | TACTAGTGCTGGTTTTCCTTT | CTCATTAACTGTGTCGTTGAT | (Kevill et al. 2017) |
| DWV-B | TACTAGTGCTGGTTTTCCTTT | CTCATTAACTGAGTTGTTGTC | (Kevill et al. 2017) |
| SBV | AACGTCCACTACACCGAAATGTC | ACACTGCGCGTCTAACATTCC | (Schurr et al. 2019) |
|  |  |  |  |
| *relish* | GCAGTGTTGAAGGAGCTGAA | CCAATTCTGAAAAGCGTCCA | (Evans et al. 2006) |
| *defensin-1* | TGTCGGCCTTCTCTTCATGG | TGACCTCCAGCTTTACCCAAA | (Li et al. 2016) |
| *defensin-2* | GCAACTACCGCCTTTACGTC | GGGTAACGTGCGACGTTTTA | (Gregorc et al. 2012) |
| *ago2* | AAAAAGAGCTATTGCGCGCT | GGTGCCCGCCTGTACATTAA | (Brutscher, Daughenbaugh, and Flenniken 2017) |
| *dicer* | TGCAGAATGAATCAAAGAACCGA | TGAGCCAATACAAAGCTGGA | (Galbraith et al. 2015) |
| *PPOact* | CGTTGAAAAGTCGAAGCAGATTAA | AGGACGCCACCGCAGTATT | (Coulon et al. 2020) |
| *vitellogenin* | AACGCCGTGAAGGTGAACAG | TATCGTAGAGAACCTCGCATTTCC | (Coulon et al. 2020) |
| *vago* | GTCATAGCGATCGTTTTCGCTG | GCTATAATACGACTCACTATAGGG CAATTAGGGAATGCAGC | (Ryabov et al. 2014) |
| *RpL32* | CGTCATATGTTGCCAACTGGTTT | CCATGAGCAATTTCAGCACAA | (Coulon et al. 2020) |
| *RpS5* | AATTATTTGGTCGCTGGAATTG | TAACGTCCAGCAGAATGTGGTA | (Boncristiani et al. 2012) |
| *DWV-B (PCR)* | CTGTAGTTAAGCGGTTATTAGAA | CTGAAGTACTAATCTCTGAG | (Ryabov et al. 2017) |
| *DWV-Rec (PCR)* | CTGTAGTTAAGCGGTTATTAGAA | CTTGGAGCTTGAGGCTCTGCA | (Ryabov et al. 2017) |

**Table B. Primers used for virus detection and immune gene expression by means of RT-qPCR and PCR.**


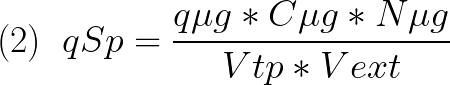

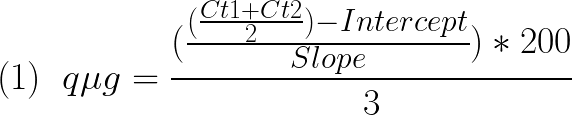

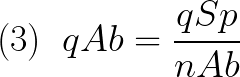


**Equations A.  Estimation of viral copies in each bee using three equations.** These equations convert the qPCR experimental data (Ct) into an estimated number of viral copies per bee. This is done by backtracking the estimated viral load using the parameters inherent to each protocol used: (1) Estimation of the number of viral copies in each µg of extracted RNA (qµg) using the qPCR experimental data (Ct1; Ct2; Intercept; Slope) and the qPCR protocol parameters (‘200’; ‘3’). ‘qµg’: estimated viral copies per µg of extracted RNA. ‘Ct1’: Quantitative cycles found for the first replicate. ‘Ct2’: Quantitative cycles found for the second replicate. ‘Intercept’ and ‘Slope’ originate from the standard curve. ‘200’ is the factor needed to convert 50ng into 1 µg. ‘3’ is the number of µg cDNA used in the qPCR protocol. (2) Estimation of the total number of viral copies present in the analysed sample using the number of viral copies in each µg of extracted RNA found in (1) (qµg), the measured RNA concentration in the sample (Cµg) and the RNA extraction protocol parameters (Nµg; Vtp; Vext). ‘qSp’: estimated viral copies in sample. ‘Cµg’: RNA concentration found in sample (µg/µL). ‘Nµg’: Volume of extracted RNA (µL). ‘Vtp’: Volume of lysis buffer used in RNA extraction (mL). ‘Vext’: Volume of supernatant transferred during the RNA extraction (mL). (3) Estimation of the viral load in each bee, using the total number of viral copies present in the sample found in (2) and the number of bee present in the sample. ‘qAb’: estimated viral copies per bee. ‘nAb’: number of bees in sample.

**Figure A. Visualisation of marked frame-bees and optical counter recording.** (a) Picture of painted and marked bees among frame bees surrounding the queen (bottom-right, painted in yellow). (b) Picture of a marked bee crossing an optical counter tunnel.


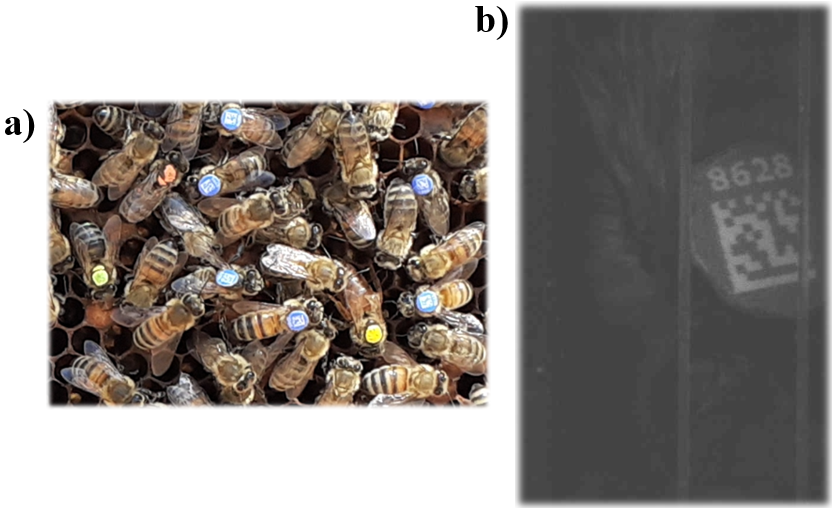


**
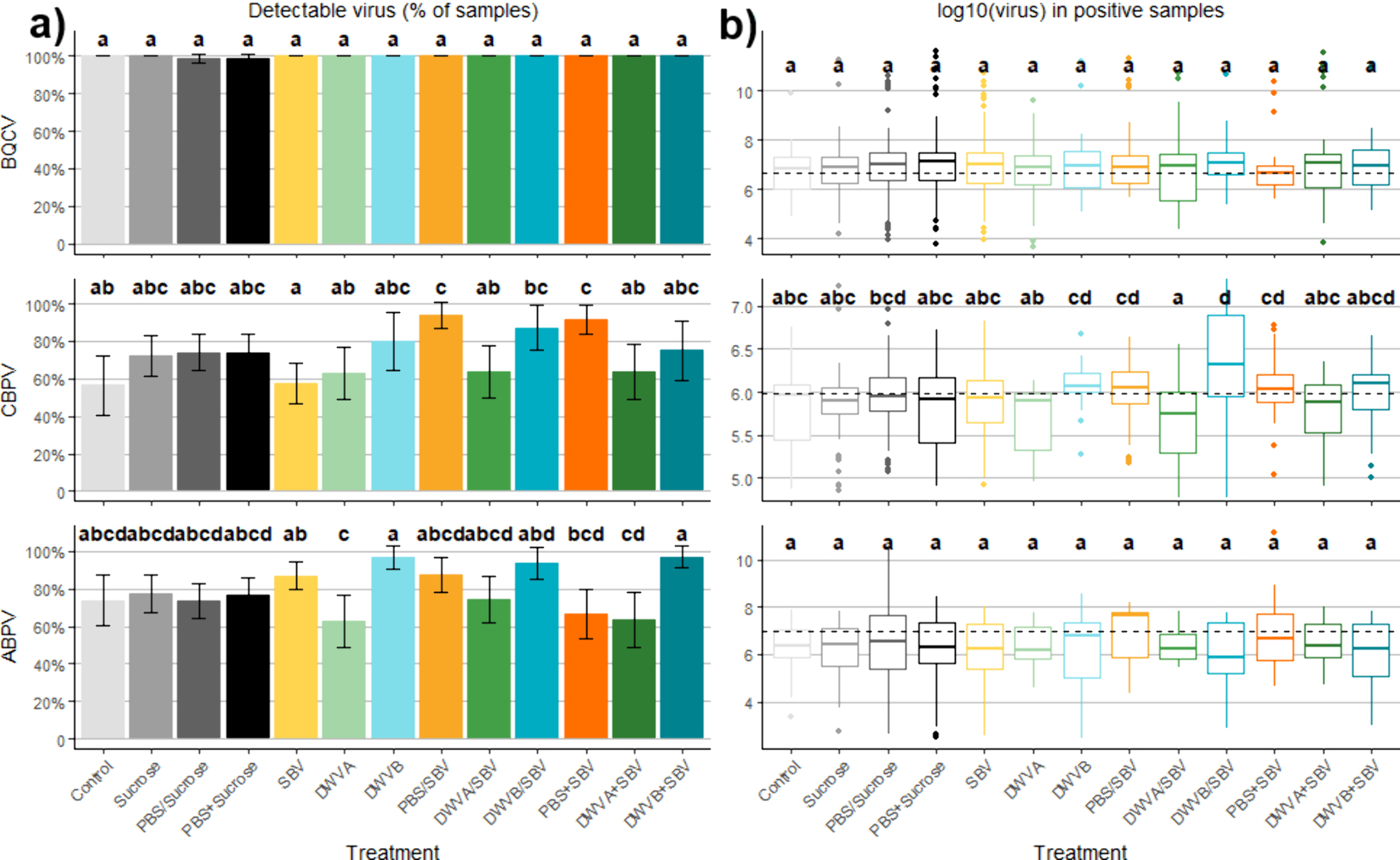
Figure B.** **BQCV, CBPV and ABPV virus detection analysis.** (a) Proportion of bees with detectable levels of virus among all sampled bees. (b) Virus loads expressed in (log_10_) in all samples with detectable levels of virus. Dotted lines represent the confidence threshold under which false positive could occur (Cq > 31). Letters represent statistically significant differences between groups for each graph ((a): pairwise nominal independence tests; (b): Dunn’s tests). Sample sizes are the same as in Figure 2 (see S1 Table).


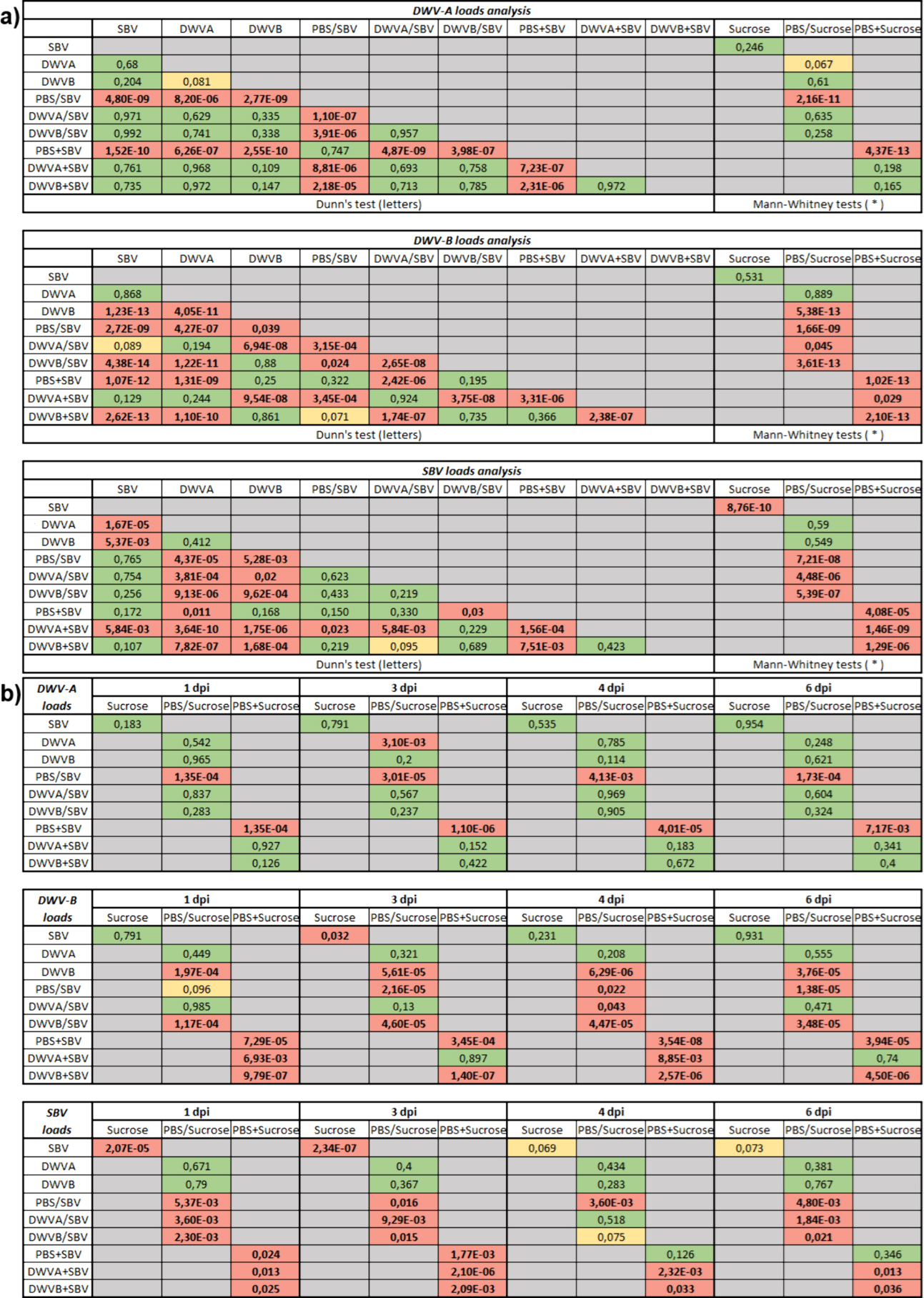
**Table C. Summary of p-values found in the statistical analysis** for (a) overall DWV-A, DWV-B or SBV loads analysis and (b) viral loads dynamics over time. The letter ‘E’ is used to signify power of 10 (e.g. 9.29E-03 is equivalent to 9.29•10^-3^). Results in bold and highlighted in red are considered statistically significant (*p* < 0.05). Results highlighted in yellow contain *p*-values comprised between 0.05 and 0.1. Results highlighted in green contain *p*-values superior to 0.1.


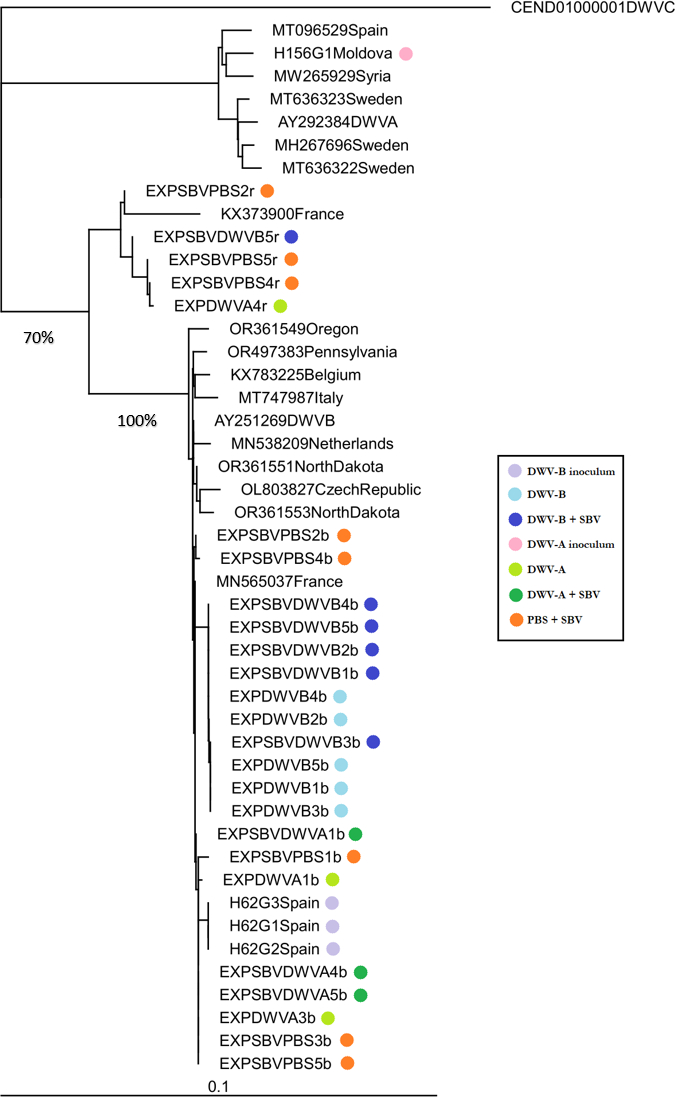
**Figure C. Phylogenetic tree of DWV sequences detected by PCR in experimental samples.** 1314 nucleotides-long sequences from the helicase coding sequence were used to construct the tree. Unmarked samples correspond to reference sequences from various European countries. Marked samples ending with “r” corresponds to PCR products using the recombinant strain primers (“DWV-Rec PCR”; S2 Table) while marked samples ending with “b” corresponds to PCR products using the DWV-B strain primers (“DWV-B PCR”; S2 Table). The phylogenetic tree was rooted using the DWV-C reference sequence as outgroup. The numbers close to the main nodes indicate the bootstrap values above 70% (1000 replicates). Bar: number of substitutions per site.

**
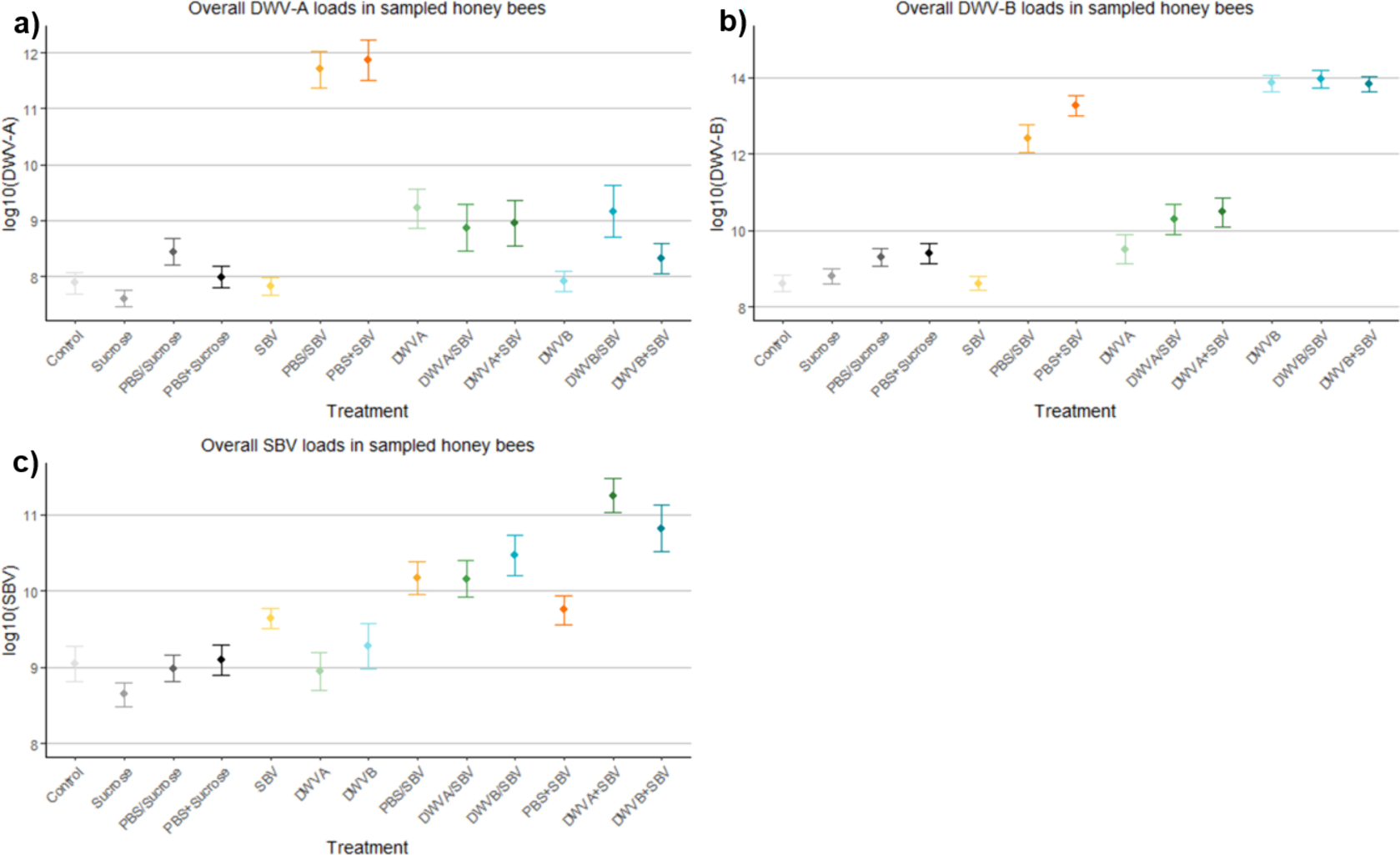
**

**Figure D. DWV-A (a), DWV-B (b) or SBV (c) loads for all treatments.** Error bars represent confidence intervals ± 95%. A “/” symbol represents sequential manipulations over two different days while a “+” symbol represents simultaneous manipulations on the same day. Sample sizes are the same as in Figure 2 (see S1 Table).

**
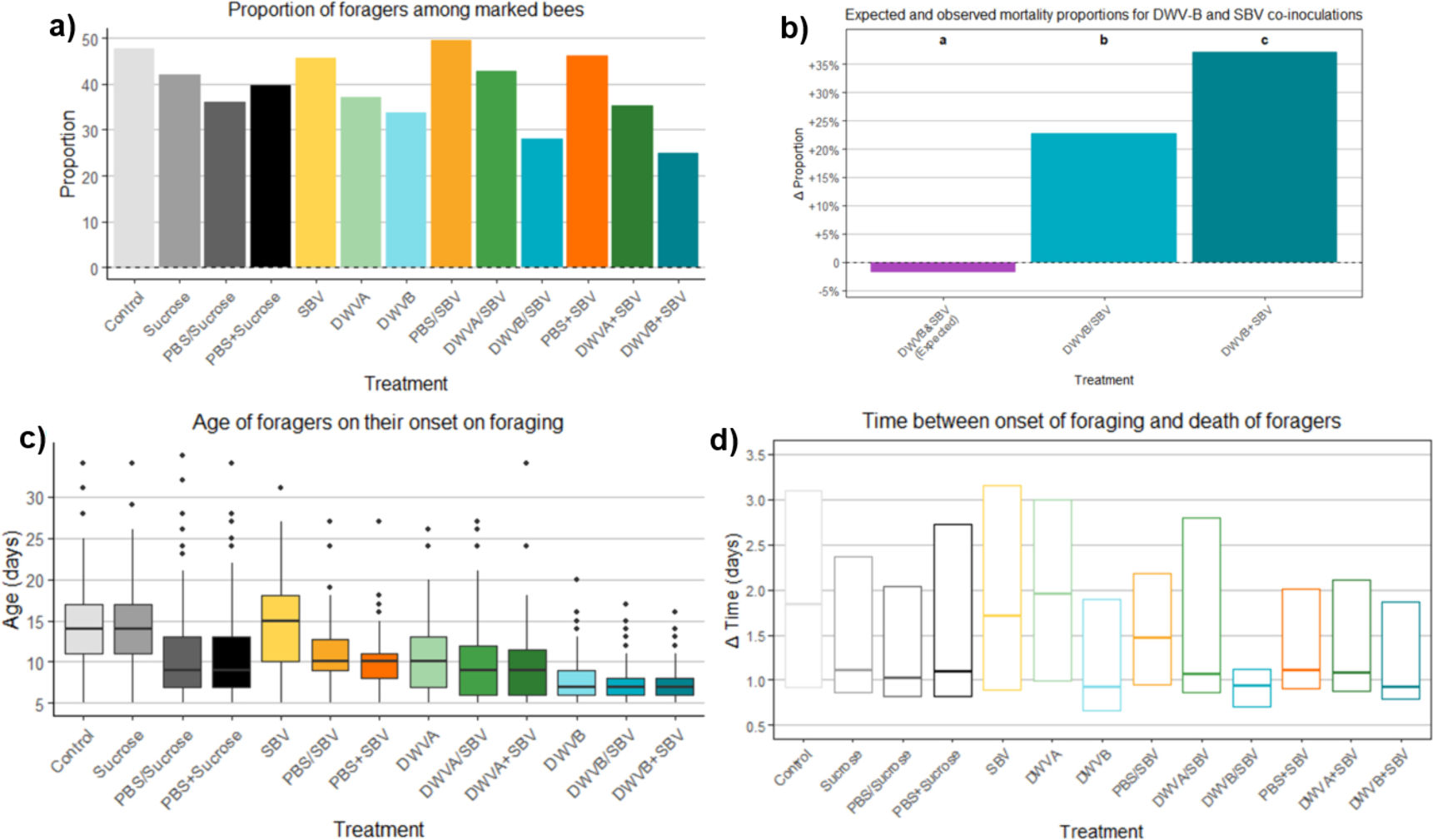
**

**Figure E. Data analysis originating from optical counters for all treatments.** (a) Proportion of marked bees that became effective foragers for all treatments. (b) Observed and expected proportions of marked bees co-inoculated with DWV-B and SBV that died before reaching their onset of foraging per cent control bees that reached their onset of foraging. Expected proportions were calculated following the Bliss independence test calculations. (c) Age at which effective foragers performed their first foraging flight. (d) Time between the onset of foraging and death of effective foragers. A “/” symbol represents sequential manipulations over two different days while a “+” symbol represents simultaneous manipulations on the same day. Sample sizes in (a) and (b) are the same as in Figure 5; samples sizes in (C) and (D) are the same as in Figure 6 (see S1 Table).

**
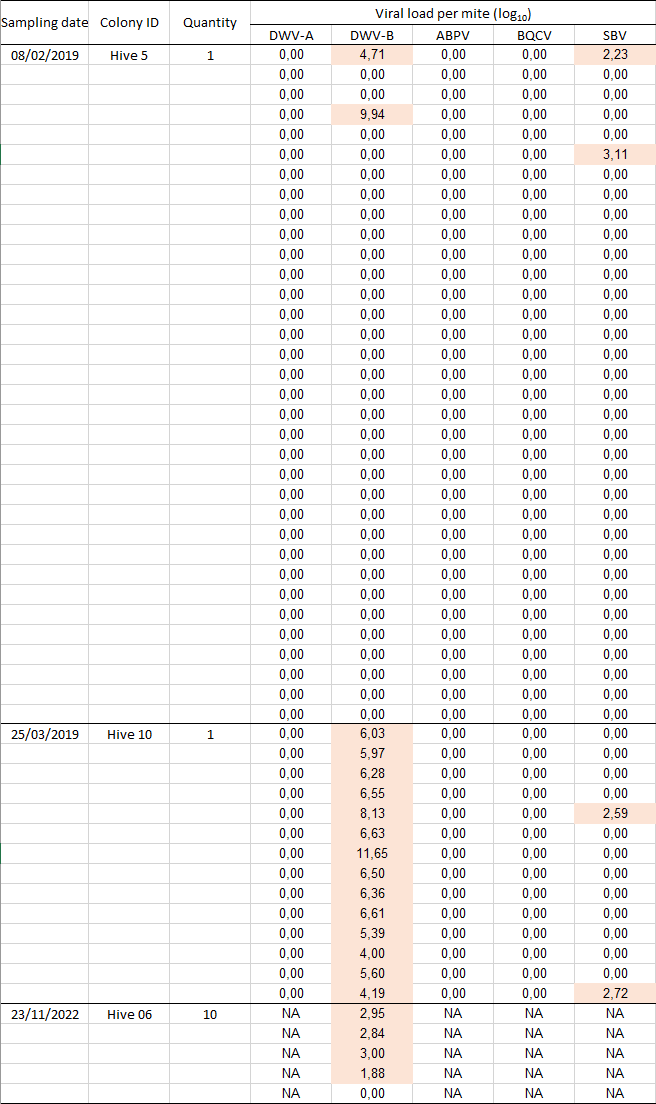
Table D.** **Viral loads of Varroa destructor mites collected throughout the year 2019 and 2021.** Viral loads of Varroa destructor mites collected on autumn 2021 (Hive 06), winter 2019 (Hive 5) and spring 2019 (Hive 10) on the apiary of ANSES Sophia Antipolis. Viral loads in mites were quantified by RT-qPCR according to the method described by (Piou et al. 2022).

**References**

Boncristiani, Humberto, Robyn Underwood, Ryan Schwarz, Jay D. Evans, Jeffery Pettis, and Dennis Vanengelsdorp. 2012. “Direct Effect of Acaricides on Pathogen Loads and Gene Expression Levels in Honey Bees Apis Mellifera.” *Journal of Insect Physiology* 58 (5): 613–20. https://doi.org/10.1016/j.jinsphys.2011.12.011.

Brutscher, Laura M., Katie F. Daughenbaugh, and Michelle L. Flenniken. 2017. “Virus and DsRNA-Triggered Transcriptional Responses Reveal Key Components of Honey Bee Antiviral Defense.” *Scientific Reports* 7 (1): 1–15. https://doi.org/10.1038/s41598-017-06623-z.

Coulon, Marianne, Anne Dalmon, Gennaro Di Prisco, Alberto Prado, Florine Arban, Eric Dubois, Magali Ribière-Chabert, Cedric Alaux, Richard Thiéry, and Yves Le Conte. 2020. “Interactions Between Thiamethoxam and Deformed Wing Virus Can Drastically Impair Flight Behavior of Honey Bees.” *Frontiers in Microbiology* 11 (April). https://doi.org/10.3389/fmicb.2020.00766.

Evans, J. D., K. Aronstein, Y. P. Chen, C. Hetru, J. L. Imler, H. Jiang, M. Kanost, G. J. Thompson, Z. Zou, and D. Hultmark. 2006. “Immune Pathways and Defence Mechanisms in Honey Bees Apis Mellifera.” *Insect Molecular Biology* 15 (5): 645–56. https://doi.org/10.1111/j.1365-2583.2006.00682.x.

Galbraith, David A., Xingyu Yang, Elina Lastro Niño, Soojin Yi, and Christina Grozinger. 2015. “Parallel Epigenomic and Transcriptomic Responses to Viral Infection in Honey Bees (Apis Mellifera).” *PLoS Pathogens* 11 (3): 1–24. https://doi.org/10.1371/journal.ppat.1004713.

Gregorc, Aleš, Jay D. Evans, Mike Scharf, and James D. Ellis. 2012. “Gene Expression in Honey Bee (Apis Mellifera) Larvae Exposed to Pesticides and Varroa Mites (Varroa Destructor).” *Journal of Insect Physiology* 58 (8): 1042–49. https://doi.org/10.1016/j.jinsphys.2012.03.015.

Kevill, Jessica L., Andrea Highfield, Gideon J. Mordecai, Stephen J. Martin, and Declan C. Schroeder. 2017. “ABC Assay: Method Development and Application to Quantify the Role of Three DWV Master Variants in Overwinter Colony Losses of European Honey Bees.” *Viruses* 9 (11): 1–14. https://doi.org/10.3390/v9110314.

Li, Wenfeng, Jay D. Evans, Qiang Huang, Cristina Rodríguez-García, Jie Liu, Michele Hamilton, Christina M. Grozinger, Thomas C. Webster, Songkun Su, and Yan Ping Chen. 2016. “Silencing the Honey Bee (Apis Mellifera) Naked Cuticle Gene (Nkd) Improves Host Immune Function and Reduces Nosema Ceranae Infections.” *Applied and Environmental Microbiology* 82 (22): 6779–87. https://doi.org/10.1128/AEM.02105-16.

Locke, Barbara, Eva Forsgren, Ingemar Fries, and Joachim R. de Miranda. 2012. “Acaricide Treatment Affects Viral Dynamics in Varroa Destructor-Infested Honey Bee Colonies via Both Host Physiology and Mite Control.” *Applied and Environmental Microbiology* 78 (1): 227–35. https://doi.org/10.1128/AEM.06094-11.

Piou, Vincent, Frank Schurr, Eric Dubois, and Angélique Vétillard. 2022. “Transmission of Deformed Wing Virus between Varroa Destructor Foundresses, Mite Offspring and Infested Honey Bees.” *Parasites and Vectors* 15 (1): 1–15. https://doi.org/10.1186/s13071-022-05463-9.

Ryabov, Eugene V., Anna K. Childers, Yanping Chen, Shayne Madella, Ashrafun Nessa, Dennis VanEngelsdorp, and Jay D. Evans. 2017. “Recent Spread of Varroa Destructor Virus-1, a Honey Bee Pathogen, in the United States.” *Scientific Reports* 7 (1): 1–10. https://doi.org/10.1038/s41598-017-17802-3.

Ryabov, Eugene V., Graham R. Wood, Jessica M. Fannon, Jonathan D. Moore, James C. Bull, Dave Chandler, Andrew Mead, Nigel Burroughs, and David J. Evans. 2014. “A Virulent Strain of Deformed Wing Virus (DWV) of Honeybees (Apis Mellifera) Prevails after Varroa Destructor-Mediated, or In Vitro, Transmission.” *PLoS Pathogens* 10 (6). https://doi.org/10.1371/journal.ppat.1004230.

Schurr, Frank, Amandine Tison, Laura Militano, Nicolas Cheviron, Fabrice Sircoulomb, Marie Pierre Rivière, Magali Ribière-Chabert, Richard Thiéry, and Eric Dubois. 2019. “Validation of Quantitative Real-Time RT-PCR Assays for the Detection of Six Honeybee Viruses.” *Journal of Virological Methods* 270: 70–78. https://doi.org/10.1016/j.jviromet.2019.04.020.

**List of supplementary information titles and legends**

**Table A. Summary of replicates and sample sizes for each treatment in our analyses.** The first table describes the total number of colonies, replicates and bees included for each treatment. The second table describes the chronology of experiments, and which treatment was included in each replicate. For Figure 4, 5 and 6, each sample consists of a pool of three honey bees. Numbers ranging over two timepoints lacked a treatment of reference (Untreated).

**Table B. Primers used for virus detection and immune gene expression by means of RT-qPCR and PCR.**

**Equations A.  Estimation of viral copies in each bee using three equations.** These equations convert the qPCR experimental data (Ct) into an estimated number of viral copies per bee. This is done by backtracking the estimated viral load using the parameters inherent to each protocol used: (1) Estimation of the number of viral copies in each µg of extracted RNA (qµg) using the qPCR experimental data (Ct1; Ct2; Intercept; Slope) and the qPCR protocol parameters (‘200’; ‘3’). ‘qµg’: estimated viral copies per µg of extracted RNA. ‘Ct1’: Quantitative cycles found for the first replicate. ‘Ct2’: Quantitative cycles found for the second replicate. ‘Intercept’ and ‘Slope’ originate from the standard curve. ‘200’ is the factor needed to convert 50ng into 1 µg. ‘3’ is the number of µg cDNA used in the qPCR protocol. (2) Estimation of the total number of viral copies present in the analysed sample using the number of viral copies in each µg of extracted RNA found in (1) (qµg), the measured RNA concentration in the sample (Cµg) and the RNA extraction protocol parameters (Nµg; Vtp; Vext). ‘qSp’: estimated viral copies in sample. ‘Cµg’: RNA concentration found in sample (µg/µL). ‘Nµg’: Volume of extracted RNA (µL). ‘Vtp’: Volume of lysis buffer used in RNA extraction (mL). ‘Vext’: Volume of supernatant transferred during the RNA extraction (mL). (3) Estimation of the viral load in each bee, using the total number of viral copies present in the sample found in (2) and the number of bee present in the sample. ‘qAb’: estimated viral copies per bee. ‘nAb’: number of bees in sample.

**Figure A. Visualisation of marked frame-bees and optical counter recording.** (a) Picture of painted and marked bees among frame bees surrounding the queen (bottom-right, painted in yellow). (b) Picture of a marked bee crossing an optical counter tunnel.

**Figure B.** **BQCV, CBPV and ABPV virus detection analysis.** (a) Proportion of bees with detectable levels of virus among all sampled bees. (b) Virus loads expressed in (log_10_) in all samples with detectable levels of virus. Dotted lines represent the confidence threshold under which false positive could occur (Cq > 31). Letters represent statistically significant differences between groups for each graph ((a): pairwise norminal independence tests; (b): Dunn’s tests). Sample sizes are the same as in Figure 2 (see S1 Table).

**Table C. Summary of p-values found in the statistical analysis** for (a) overall DWV-A, DWV-B or SBV loads analysis and (b) viral loads dynamics over time. The letter ‘E’ is used to signify power of 10 (e.g. 9.29E-03 is equivalent to 9.29•10^-3^). Results in bold and highlighted in red are considered statistically significant (*p* < 0.05). Results highlighted in yellow contain *p*-values comprised between 0.05 and 0.1. Results highlighted in green contain *p*-values superior to 0.1.

**Figure C. Phylogenetic tree of DWV sequences detected by PCR in experimental samples.** 1314 nucleotides-long sequences from the helicase coding sequence were used to construct the tree. Unmarked samples correspond to reference sequences from various European countries. Marked samples ending with “r” corresponds to PCR products using the recombinant strain primers (“DWV-Rec PCR”; S2 Table) while marked samples ending with “b” corresponds to PCR products using the DWV-B strain primers (“DWV-B PCR”; S2 Table). The phylogenetic tree was rooted using the DWV-C reference sequence as outgroup. The numbers close to the main nodes indicate the bootstrap values above 70% (1000 replicates). Bar: number of substitutions per site.

**Figure D. DWV-A (a), DWV-B (b) or SBV (c) loads for all treatments.** Error bars represent confidence intervals ± 95%. A “/” symbol represents sequential manipulations over two different days while a “+” symbol represents simultaneous manipulations on the same day. Sample sizes are the same as in Figure 2 (see S1 Table).

**Figure E. Data analysis originating from optical counters for all treatments.** (a) Proportion of marked bees that became effective foragers for all treatments. (b) Observed and expected proportions of marked bees co-inoculated with DWV-B and SBV that died before reaching their onset of foraging per cent control bees that reached their onset of foraging. Expected proportions were calculated following the Bliss independence test calculations. (c) Age at which effective foragers performed their first foraging flight. (d) Time between the onset of foraging and death of effective foragers. A “/” symbol represents sequential manipulations over two different days while a “+” symbol represents simultaneous manipulations on the same day. Sample sizes in (a) and (b) are the same as in Figure 5; samples sizes in (C) and (D) are the same as in Figure 6 (see S1 Table).

**Table D.** **Viral loads of Varroa destructor mites collected throughout the year 2019 and 2021.** Viral loads of Varroa destructor mites collected on autumn 2021 (Hive 06), winter 2019 (Hive 5) and spring 2019 (Hive 10) on the apiary of ANSES Sophia Antipolis. Viral loads in mites were quantified by RT-qPCR according to the method described by (Piou et al. 2022).
